# Effect of Cilostazol in the Expression of Biomarkers and Neurological Outcome Following Experimentally Induced Cerebrovascular Accident—Experimental Protocol

**DOI:** 10.3390/neurolint17080126

**Published:** 2025-08-11

**Authors:** Christiana Anastasiadou, Stavroula Kastora, Alkistis Kapelouzou, Anastasios Papapetrou, Angelos Megalopoulos, Nikolaos Kostomitsopoulos, Efthymios Paronis, Andreas Lazaris, George Geroulakos, Christos Liapis, Nikolaos Saratzis, John Kakisis

**Affiliations:** 1 Department of Vascular Surgery, “George Papanikolaou” General Hospital of Thessaloniki, 57010 Exochi, Greece; megalangelo@yahoo.com; 2 EGA Institute for Women’s Health, University College London, London WC1E 6BT, UK; s.kastora@ucl.ac.uk; 3 Clinical, Experimental Surgery & Translational Research, Biomedical Research Foundation Academy of Athens, 11527 Athens, Greece; akapel@bioacademy.gr (A.K.); nkostom@bioacademy.gr (N.K.); eparonis@bioacademy.gr (E.P.); 4 Department of Vascular Surgery, “KAT” General Hospital of Athens, 14561 Kifisia, Greece; tas1sos@yahoo.gr; 5 Department of Vascular Surgery, “Attikon” University Hospital, School of Medicine, National and Kapodistrian University of Athens, 10679 Athens, Greece; andreaslazaris@hotmail.com (A.L.); ggeroulakos@med.uoa.gr (G.G.); liapis@med.uoa.gr (C.L.); kakisis@med.uoa.gr (J.K.); 6 Department of Vascular Surgery, “G. Papageorgiou” University Hospital of Thessaloniki, 56429 Pavlos Melas, Greece; nicos_saratzis@yahoo.com

**Keywords:** infarction, middle cerebral artery, cilostazol, aspirin, biomarkers

## Abstract

Objective: Several strategies have been described for stroke prevention, and the most commonly used medication is aspirin. Cilostazol, which is a substance with a pleiotropic effect, is still not well investigated. In this study, we aimed to delineate the effects of mono- and combinatorial pre-treatment upon neurological status and biomarkers, namely protein S100b, GFAP, procalcitonin, and galectin-3, following stroke. Methods: Twelve-week-old Sprague–Dawley rats were randomly assigned to four groups, each containing six rats: control group (normal saline), cilostazol group (30 mg/kg/daily), aspirin group (10 mg/kg/daily), and aspirin/cilostazol group. Each substance was administered by gavage for four weeks. All animals were subjected to cerebral ischemia for 2 h using intraluminal middle cerebral artery occlusion. A neurological examination was performed, serum concentrations of biomarkers were determined, and the animals were then sacrificed. Results: All treatment groups exhibited variations in the severity of immediate neurological presentation. Unlike the control group, where all rats presented with severe focal neurology or mortality, most rats in the treatment groups displayed no to moderate focal neurology. Moreover, the aspirin/cilostazol group consistently exhibited significantly lower levels in the studied biomarkers compared to other groups. Conclusions: Co-administration of cilostazol and aspirin significantly ameliorates the immediate expression of the studied biomarkers. Further large-scale studies are needed to investigate the effect of combined therapy for primary and secondary prevention of stroke, using not only serum biomarkers but other specific clinical and laboratory endpoints.

## 1. Introduction

Several strategies have been described for cerebrovascular accident (CVA) prevention, including behavioral modifications such as smoking cessation, increased physical activity, and healthy diet, as well as medical and open surgical/endovascular interventions. Regarding medical management, aspirin and statins are considered to be the golden standard for the pharmacological prevention of cerebrovascular accidents as they have displayed well-established protective effects upon the vasculature [1]. Aspirin serves as an antiplatelet agent, lowering the risk of thrombotic events. Statins are lipid-lowering medications that also stabilize atherosclerotic plaques. Similarly, cilostazol, a substance with pleiotropic effects, could theoretically contribute to non-invasive prevention of permanent cerebral ischemia as it possesses antiplatelet, vasodilatory and anti-inflammatory effects [2].

Regarding blood biomarkers, they could be used for several purposes: to aid in diagnosis and uncover stroke etiology, or to monitor treatment efficacy and identify the risk of complications. Several blood biomarkers are being investigated for their use in CVAs. Among brain-enriched proteins, glial fibrillary acidic protein (GFAP) and s-100b calcium binding protein are the most used, as they increase early after stroke and are associated with extent of brain injury and the degree of disability [3,4,5,6,7,8]. Likewise, GFAP is reported to add in diagnosis as it distinguishes ischemic from hemorrhagic stroke and S100b predicts hemorrhagic transformation [9]. Galectin-3 seems to participate in the atherosclerotic process and to enhance the recruitment of inflammatory cells to the atherosclerotic plaque [10]. Both galectin-3 and procalcitonin are reported to be associated with stroke severity and poor outcomes [10,11,12,13,14,15,16,17]. While previous studies have highlighted the favorable effect of cilostazol in white matter, blood–brain barrier, and infarct volume [18,19,20,21,22], the pre-treatment with cilostazol for four weeks and its effect on the specific range of biomarkers has never been described before. In the present work, we aimed to delineate the effects of mono- and combinatorial pre-treatment upon neurological status and neurological integrity biomarkers, namely protein S100b, GFAP, procalcitonin, and galectin-3, following a cerebrovascular accident. We selected both astroglial (GFAP, S100b) and systemic inflammatory (galectin-3, procalcitonin) markers to capture complementary pathophysiological phases after permanent cerebral ischemia. Previous work demonstrates that GFAP rises within 60 min of injury, whereas S100b and procalcitonin peak later (6–24 h). Sampling at 120 min therefore permitted an early yet integrative snapshot while minimizing mortality in this severe model.

## 2. Materials and Methods

Male Sprague–Dawley healthy rats, 12 weeks old, were randomly allocated into four groups (n = 6 per group): control (receiving saline), cilostazol (30 mg/kg/day), aspirin (10 mg/kg/day), and a combination group (aspirin + cilostazol). Treatments were administered via oral gavage daily for four weeks before inducing permanent ischemia. Following pre-treatment, all animals underwent permanent focal cerebral ischemia for two hours using the intraluminal middle cerebral artery occlusion (MCAO) method.

Each animal then received a standardized neurological evaluation. Blood samples were collected to measure serum levels of galectin-3, procalcitonin, GFAP, and protein S100b. Finally, animals were euthanized in accordance with ethical guidelines. The selected doses are consistent with those commonly employed in preclinical stroke models and reflect pharmacokinetic properties established in earlier studies.

Ethical approval was granted by the Scientific Committee for the Approval of Protocols Using Animals for Scientific Purposes at the Biomedical Research Foundation, Academy of Athens (Laboratory for Experimental Surgery and Surgical Research) and by the Veterinary Directorate of the Attica Region (AΠ:602325/08.10.2019).

Sample size adequacy was determined using a resource equation approach (details provided in Supplementary Materials). Post hoc analysis based on group means and standard deviations confirmed the statistical soundness of our design.

Prior to surgery, animals were fasted for 12 h with ad libitum access to water. Anesthesia was induced with inhaled isoflurane (2.0–3.0 mL/L), and animals were placed supine on a surgical platform before undergoing permanent MCAO.

### 2.1. Surgical Procedure

Left common, internal and external carotid arteries were exposed and carefully dissected, avoiding injury to soft tissues and nerves. The internal carotid artery (ICA) was isolated and carefully separated from the adjacent vagus nerve. The external carotid artery (ECA) was ligated distally with a 6-0 silk suture. Another 6-0 silk suture was placed loosely around the ECA near the bifurcation with the ICA, being careful not to occlude the vessel. Microsurgical clamps were placed on the common and internal carotid artery near the bifurcation and the ECA was partially incised between the 2 silk suture ties. A silicone rubber-coated monofilament suture Large MCAO (Doccol Corporation, Sharon, MA, USA) 30 mm in length with a 1–2 mm silicone-coated tip (0.41 mm diameter) was advanced into the ECA lumen. After temporarily clamping the internal carotid artery (ICA), a nylon monofilament was gently introduced through the external carotid artery (ECA) and advanced into the ICA lumen. The filament was inserted until mild resistance was encountered, indicating occlusion at the origin of the middle cerebral artery (MCA) [23]. The duration of occlusion was recorded. Once the procedure was completed, the incision was sutured in layers and animals were transferred to a recovery area. They were closely monitored postoperatively for signs of pain, distress, or procedural complications. Neurological function was evaluated, and any adverse outcomes were promptly addressed with appropriate veterinary care. Detailed records were maintained for each animal, including any postoperative observations. After 120 min of sustained ischemia, blood was collected via pericardiocentesis, followed by humane euthanasia in accordance with institutional protocols.

### 2.2. Neurologic Examination

Following full recovery from anesthesia, each animal underwent standardized neurological evaluation. Motor deficits and neurological impairment were assessed using the Longa scoring system, a five-point scale frequently employed in rodent stroke models. The scale ranges from 0 to 4:

- 0: No observable neurological deficit
- 1: Incomplete extension of the contralateral forelimb (mild deficit)
- 2: Consistent circling toward the contralateral side (moderate impairment)
- 3: Spontaneous circling even at rest (severe motor impairment)
- 4: Absence of spontaneous movement or loss of consciousness (profound deficit)

All personnel involved in scoring were extensively trained in the Longa system to ensure reliable and reproducible assessment of neurological function.

### 2.3. Blood Collection—ELISA Measurements

Blood samples were collected via pericardiocentesis after 120 min of ischemia to capture the biomarkers at the desired time point. We then transferred the collected blood into sterile centrifuge tubes and allowed it to clot at room temperature for 30 min. Subsequently, we centrifuged the samples at 1500–2000× *g* for 10–15 min at room temperature to separate the serum from the cellular components. We then transferred the serum to new sterile tubes without disturbing the pellet. The serum samples that were collected were immediately frozen at −80 °C to preserve the integrity of the biomarkers. Samples were maintained at −80 °C until analysis to ensure stability and reproducibility of results. ELISA rat kits were utilized for the following biomarkers: Procalcitonin (Abbexa, Cambridge, UK, abx255900), galectin-3 (Invitrogen, Thermo Fisher Scientific, Waltham, MA, USA, ERLGALS3), protein S100b (Cusabio, Houston, TX, USA, CSB E08066r), and GFAP (Cusabio, USA, CSB-E08602r). Serum biomarkers were analyzed using an ELISA reader system (Spectramax 190; Molecular Devices, Sunnyvale, CA, USA). S100b Protein B and GFAP were chosen as markers specifically associated with the central nervous system, whereas galectin 3 and procalcitonin were chosen as biomarkers associated with various physiological processes and conditions. In the supplementary files, one can find the intra- and inter-assay provided by the manufacturers of the ELISA kits. However, we shall mention that all the ELISA kits were from the same batch (per marker) and therefore intra-assay variability was not required. Intra-assay coefficients of variation (CVs) were 4.6–6.2 % and inter-assay CVs were <8 %, as verified by duplicate quality-control samples and considered negligible (Table S1).

Technical replicates were represented and accounted for 95% confidence intervals in cumulative result graphs. Inter-assay variability assessment could not be carried out as that assays are relevant to different markers and thus fundamentally non-comparable.

### 2.4. Statistical Analysis

Sample size justification: We first applied the resource equation (E = N–G) aiming for 10 ≤ E ≤ 20. With N = 24 and G = 4, E = 20 met the upper acceptable limit. A priori power analysis for our primary outcome (serum S100b) used pilot data (σ = 0.21 ng/mL, Δ = 0.30 ng/mL) with α = 0.05 and β = 0.20, giving n = [(1.96 + 0.84)^2^ × 2σ^2^]/Δ^2^ = 5.4 rats per group; we rounded up to six.

The treatment and control groups were independent and normally distributed. Therefore, for two sample comparison, independent t-test was deemed appropriate, whilst for multigroup analysis, ANOVA was chosen. Intergroup variance assessment satisfied the equality assumption between groups. As a unidirectional effect was anticipated (cilostazol ± aspirin would lower biomarker levels), one-tailed independent-samples t-tests were planned. Effect sizes (Cohen’s d) accompany all *p* values. Data met Shapiro–Wilk normality and Levene homogeneity criteria; otherwise, Kruskal–Wallis and Dunn’s tests were used. Data are expressed as mean and standard deviation (SD). All analyses were carried out using software Graph Pad Prism program version 10. Cut-off for statistical significance was set at *p* < 0.05.

## 3. Results

**Figure 1:**
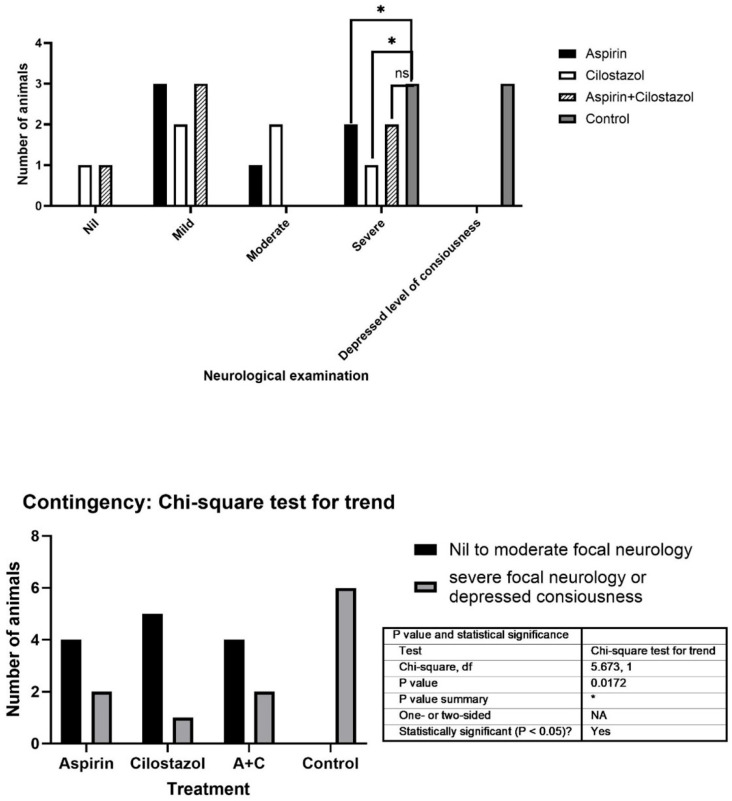
Neurologic examination among the groups. (* indicates statistical significance (*p* < 0.05), while “ns” stands for “not significant").

Twenty-four rats were randomly distributed into four groups, each consisting of six rats: the control group (CON), cilostazol group (C), aspirin group (A), and aspirin/cilostazol group (AC). Regarding the initial clinical manifestation after stroke, the neurologic deficit score among the groups is presented in Figure 1. In the CON group, three deaths were observed shortly before the completion of the 120 min ischemic period. All rats of the remaining groups survived (Figure 1).

**Figure 2:**
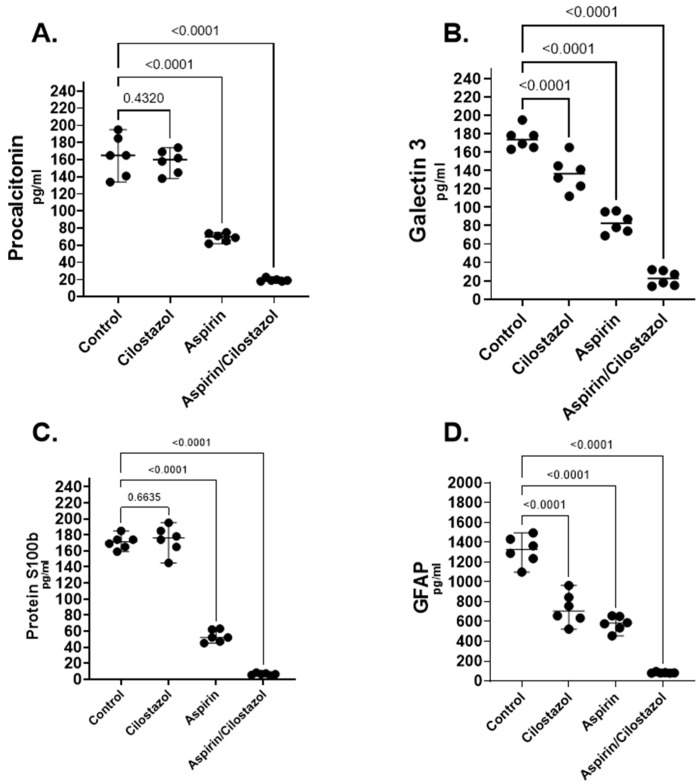
Serum biomarker analysis of procalcitonin (**A**), galectin-3 (**B**), protein S100b (**C**), and glial fibrillary acidic protein (GFAP) (**D**) in the control group, cilostazol group, aspirin group, and aspirin/cilostazol group. Data were analyzed to assess the effects of individual and combined therapies on biomarker expression following permanent cerebral injury.

Considering serum biomarkers, the values from all the controls are presented in Table 1. Compared to the rats in the other groups, the aspirin/cilostazol group had significantly lower levels of GFAP. Median values for the various groups were as follows: CON 1327 pg/mL, C 707 pg/mL, A 583.5 pg/mL, AC 81.5 pg/mL (*p* < 0.0001). The cilostazol group did not differ significantly from the aspirin group. All medication groups differ significantly when compared to the control group (Figure 2D and Figure S2).

Regarding S100b, the levels in the aspirin/cilostazol group were significantly lower than in the other groups. Median values for the four groups were as follows: CON 171.5 pg/mL, C 176 pg/mL, A 52 pg/mL, AC 6.3 pg/mL (*p* < 0.0001). The group with the second lowest levels of S100b was the aspirin group. The cilostazol group did not have any difference compared to control group (Figure 2 and Figure S2).

Considering galectin-3 levels, the aspirin/cilostazol group showed markedly lower levels than the other groups. The group with the second lowest levels was the aspirin group (Figure 2B and Figure S2). Median values for the four groups were as follows: CON 173.5 pg/mL, C 136.3 pg/mL, A 83.17 pg/mL, AC 22.83 pg/mL (*p* < 0.0001).

Regarding procalcitonin levels, the aspirin/cilostazol group exhibited significantly lower levels than the other groups. Median values for the four groups were as follows: CON 165 pg/mL, C 160 pg/mL, A 70 pg/mL, AC 19 pg/mL (*p* < 0.0001). The cilostazol group did not have any difference compared to the control group, and the group with the second lowest levels of procalcitonin was the aspirin group in this case as well (Figure 2A and Figure S2).

## 4. Discussion

This experimental study investigated the effects of cilostazol, aspirin, and their co-administration on immediate neurological status and serum biomarkers in rats following permanent cerebral injury. Unlike previous studies, which examined the individual effects of each substance, long-term outcomes, or different models of cerebral ischemia (such as transient cerebral ischemia), our study focused on the immediate impact in a permanent injury model. In this experimental protocol, our aim was to assess the clinical and laboratory effects of adding cilostazol to the standard regimen of aspirin for primary or secondary stroke prevention. We observed that cilostazol significantly affected the expression of biomarkers, especially in the combinatorial pre-treatment. Therefore, we believe that cilostazol is a pharmacological agent worth examining in a broader sample, including patients with carotid artery occlusion that are not amenable to surgical repair, as well as in patients after cerebrovascular accidents (CVA). Beyond its role in stroke prevention, cilostazol’s vasodilatory, anti-inflammatory, and endothelial-protective properties may also offer benefits in reducing the risk of silent cerebral infarctions, which may contribute to cognitive decline and vascular dementia. Improving microcirculatory flow cilostazol, in combination with aspirin, could provide an important treatment strategy for both stroke prevention and long-term neuroprotection. Future clinical trials should explore its potential in these populations to better define its role in cerebrovascular health and cognitive preservation.

Following a cerebrovascular accident, there is an inflammatory response and activation of glial cells, including astrocytes. Astrocytes are cells that provide structural support to neurons, regulate the extracellular environment and participate in synaptic transmission and repair processes [24]. Glial fibrillary acidic protein, abbreviated as GFAP, as well as S100b, are primarily found in cells of central nervous system [4]. In this context, several studies have investigated these proteins as potential biomarkers for stroke. Both GFAP and S100B serve as promising biomarkers for assessing the severity and prognosis of stroke [3,4,7,8,12,25,26]. Our study showed that the aspirin/cilostazol group presented significantly lower levels in both biomarkers. The group receiving aspirin exhibited lower levels than either the control or cilostazol groups, yet its levels significantly differed from those observed in the combined therapy group. Regarding S100b, the cilostazol group did not differ from the control group. This could be probably associated with the timing of blood sampling. S100b levels are higher at least 48 h after the onset of cerebral ischemia, whereas GFAP levels increase sooner after ischemia onset, making it a more suitable biomarker for early diagnosis. However, we cannot interpret the finding that in the combined therapy group, levels are significantly lower than that of cilostazol [7].

Similarly, other biomarkers that are not specific to CNS are being investigated to determine their suitability for use as biomarkers for CVAs. Following cerebral ischemia, gal-3 is released by activated microglia and astrocytes, contributing to the inflammatory cascade [12,27]. The activation of immune cells releases cytokines and reactive oxygen species. Studies in humans show that gal-3 is associated with stroke severity and infarct volume, as well as overall poor outcomes [10,12,26]. In our study, when examining gal-3 levels, the combined therapy group exhibited significantly lower levels compared to all other groups. Procalcitonin has also been utilized as a biomarker since it may suggest the occurrence of secondary infections, such as pneumonia or urinary tract infection [15,16]. Furthermore, an association between elevated procalcitonin levels and malignant cerebral oedema resulting in CVA unfavorable outcomes has been previously reported [15]. In terms of procalcitonin levels, the aspirin/cilostazol group showed significantly lower levels than the other groups. The cilostazol group displayed no discernible difference compared to the control group, similar to what was observed with S100b. Like S100b, procalcitonin is a biomarker whose levels rise within the first 24–72 h.

In our study, all treatment groups exhibited variations in the severity of immediate neurological presentation. Unlike the control group, where all rats presented with severe focal neurology or mortality, most rats in the treatment groups displayed no to moderate focal neurology. Moreover, the aspirin/cilostazol group consistently exhibited significantly lower levels in the studied biomarkers compared to other groups. Therefore, there is a synergistic effect of cilostazol with aspirin, that may impact upon the serological levels of those biomarkers.

Aspirin acts as a COX enzymes inhibitor, thereby reducing inflammation and preventing blood clot formation. In contrast cilostazol acts by inhibiting phosphodiesterase III enzyme, thereby increasing cAMP levels, leading to vasodilation and inhibiting platelet aggregation [28]. The precise mechanism through which cilostazol elicits its beneficial effects are not comprehensively understood. A captivating study revealed that subjecting brain endothelial cells to oxygen–glucose deprivation and subsequent reoxygenation markedly triggered endoplasmic reticular stress while impairing the barrier function of cell monolayers. Moreover, administration of cilostazol mitigated the stress induced by oxygen–glucose deprivation and preserved barrier function [29]. Yet another study suggested that cilostazol might enhance autoregulatory responses within cortical cerebral arteries by upregulating eNOS phosphorylation and VEGF expression in individuals with diabetes. Furthermore, cilostazol could potentially serve as a neurovascular protectant in this patient population [30]. Whilst adequately powered randomized control trials, with long enough follow-up periods, to assess the effect of cilostazol in CVA patients have not yet been conducted, multiple observational studies have verified the positive effect of cilostazol in patients with stroke [2,20,24,25,26,27,28,29,30,31,32,33].

## 5. Limitations of the Study

Our study was subjected to several limitations. Firstly, peak serum levels of biomarkers may not occur immediately after cerebral ischemia and in turn our sampling is subject to peak biomarker release which does not coincide across all biomarkers. However, a set, single time point was selected for consistency of measurements. Specifically, for S100b and procalcitonin in humans, several days may be required for the highest levels to be observed, in order to correlate them with specific clinical outcomes. Additionally, multiple measurements of a biomarker are necessary to establish its clinical utility. This allows better assessment of biomarker’s correlation with clinical outcomes and enhances the statistical power. Unfortunately, with a permanent MCAO ischemia, the mortality is quite high, estimated to be 20–50% in 24 h [34]. As the possibility to lose a considerable number of lab rats was notable, we opted for a single biomarker measurement instead. Finally, one should note that the biomarkers’ value must be considered in conjunction with other diagnostic tests (e.g., magnetic resonance imaging) or functional tests (such as Rotarod test, sticky tape test, beam walk test) to measure the extent of brain tissue damage and that these biomarkers are not CVA-specific markers, as their levels are increased in serum even after trauma.

## 6. Conclusions

Our study demonstrated that combined administration of cilostazol and aspirin significantly modulates serum biomarker levels associated with cerebral ischemia, suggesting potential neuroprotective effects. However, the impact of this combined therapy on neurological function, behavioral outcomes, and long-term recovery remains to be fully elucidated. Further large-scale clinical studies are needed to investigate the effect of combined therapy for primary and secondary prevention of stroke, using not only serum biomarkers but also other specific clinical and laboratory endpoints, such as MRI findings, functional outcomes, and quality of life to provide a more comprehensive understanding of the therapy’s impact.

## Figures and Tables

**Table 1:** Summary of biomarker levels (approx. mean ± SD) and effect sizes.

Biomarker	Group	Mean (pg/mL)	SD	95% CI	ANOVA *p*	Cohen’s d vs. CON	n
**GFAP**	CON	1327	331.8	978.6–1675.4	<0.0001	–	6
**GFAP**	C	707	176.8	521.4–892.6		−2.33	6
**GFAP**	A	583.5	145.9	430.3–736.7		−2.9	6
**GFAP**	AC	81.5	20.4	60.1–102.9		−5.3	6
**S100b**	CON	171.5	42.9	126.5–216.5	<0.0001	–	6
**S100b**	C	176	44.0	129.8–222.2		0.1	6
**S100b**	A	52	13.0	38.4–65.7		−3.77	6
**S100b**	AC	6.3	1.6	4.6–8.0		−5.44	6
**Galectin-3**	CON	173.5	43.4	127.9–219.1	<0.0001	–	6
**Galectin-3**	C	136.3	34.1	100.5–172.1		−0.95	6
**Galectin-3**	A	83.17	20.8	61.3–105.0		−2.65	6
**Galectin-3**	AC	22.83	5.7	16.8–28.8		−4.87	6
**Procalcitonin**	CON	165	41.2	121.7–208.3	<0.0001	–	6
**Procalcitonin**	C	160	40.0	118.0–202.0		−0.12	6
**Procalcitonin**	A	70	17.5	51.6–88.4		−3.0	6
**Procalcitonin**	AC	19	4.8	14.0–24.0		−4.98	6

## Data Availability

You can contact the author to ask for the original data based on reasonable request.

## Supplementary Materials

The following supporting information can be downloaded at: https://www.mdpi.com/article/10.3390/neurolint17080126/s1, Figure S1: Serum analysis of Glial fibrillary acidic protein, Galectin 3, Procalcitonin and Protein s100b. Data is presented as mean ± SD; Figure S2: Tukey’s Multiple Comparison Test. Statistical significance *p* < 0.05; Table S1: ELISA kit performance characteristics.
