# Supplementary material for: Effect of Cilostazol in the Expression of Biomarkers and Neurological Outcome Following Experimentally Induced Cerebrovascular Accident—Experimental Protocol
Source: Neurol Int. 2025 Aug 11;17(8):126. doi: 10.3390/neurolint17080126 (PMC12388862; doi:10.3390/neurolint17080126)
Supplement: Supplementary file 1 [file neurolint-17-00126-s001.zip › neurolint-3722563-supplementary.pdf]

Biomarkers	Groups	CON	C	A	AC
	No	6	6	6	6
GFAP	mean	1320	730.5	578.2	83.83
	SD	142.6	158.6	75.69	5.77
Galectin 3	mean	174.7	136.3	83.17	22.83
	SD	11.81	18.48	11.23	8.13
Procalcitonin	mean	164.2	157.7	69.33	19.5
	SD	23.8	13.87	5.08	1.87
Protein s100b	mean	171	173.7	53.5	6.31
	SD	8.92	17.32	7.5	1.33

Figure S1. Serum analysis of Glial fibrillary acidic protein, Galectin 3, Procalcitonin and Protein s100b. Data is presented as mean±SD.

Tukey's Multiple Comparison Test	GFAP	Galectin 3	s100b	Procalcitonin
	$p < 0.05$	$p < 0.05$	$p < 0.05$	$p < 0.05$
CON vs C	Yes	Yes	No	No
CON vs A	Yes	Yes	Yes	Yes
CON vs AC	Yes	Yes	Yes	Yes
C vs A	No	Yes	Yes	Yes
C vs AC	Yes	Yes	Yes	Yes
A vs AC	Yes	Yes	Yes	Yes

Figure S2. Tukey's Multiple Comparison Test. Statistical significance $p<0.05$.

Biomarker	Vendor	Catalogue No.	Detection range (pg/mL)	Sensitivity (pg/mL)	Intra-assay CV (%)	Inter-assay CV (%)
Procalcitonin	Abbexa	abx255900	15.6-1000	9.4	<10	<12
Galectin-3	Invitrogen	ERLGALS3	3.28-800	3	<10	<12
S100b	Cusabio	CSB-E08066r	3.12-200	0.78	<8	<10
GFAP	Cusabio	CSB-E08602r	78-5000	19.5	<8	<10

Table S1. ELISA kit performance characteristics
